# Herpes Simplex Virus 1 Encephalitis in First Trimester of Pregnancy: A Case Report

**DOI:** 10.1155/crog/8743269

**Published:** 2026-04-08

**Authors:** Matej Furlan, Miha Lučovnik, Nina Grasselli Kmet

**Affiliations:** ^1^ Infectious Diseases Department, University Medical Centre, Ljubljana, Slovenia, kclj.si; ^2^ Divison of Gynaecology and Obstetrics, University Medical Centre, Ljubljana, Slovenia, kclj.si; ^3^ Faculty of Medicine, University of Ljubljana, Ljubljana, Slovenia, uni-lj.si

**Keywords:** encephalitis, herpes simplex virus 1, high-dose acyclovir, pregnancy, therapeutic termination of pregnancy

## Abstract

Encephalitis caused by herpes simplex virus 1 (HSV‐1) has been described in pregnancy, but it’s rare, with less than 20 cases being described in peer‐reviewed literature. Physiologic changes in immune response during pregnancy influence the course of HSV‐1 encephalitis (HSVE) and predispose pregnant women to severe complications. We present a case of herpetic encephalitis in a 12‐weeks pregnant patient. In our patient neurologic condition deteriorated despite early diagnosis, appropriate antiviral and antiepileptic treatment, and suppurative neurocritical care, respectively. Disease progression stopped and the patient’s condition improved after pregnancy termination. Improvement could be a consequence of multiple factors, including delayed therapeutic effect of antiviral treatment, the impact of intensive care management, seizure control, and the possibility of spontaneous recovery as part of the natural disease course, respectively. This case highlights the potential severity of HSV‐1 in early pregnancy and underscores the importance of multidisciplinary management and individualized decision‐making in complex clinical situations.

## 1. Introduction

Herpes simplex virus (HSV) is a common cause of encephalitis, with the majority of cases due to HSV‐1; up to 50% of patients require treatment in the intensive care unit (ICU). Early treatment reduces mortality from 70% to 20%–30% [1, 2].

HSV encephalitis (HSVE) has been reported during pregnancy, but data on its clinical presentation and outcomes are limited. Dodd et al. [3] reviewed reported cases until 2015, noting that most occurred in the 2^nd^ and 3^rd^ trimester of pregnancy. To date, only one case has been reported in the first trimester [3–5]. Pregnancy is a recognized state of relative immunodeficiency due to complex immunological adaptations that prevent rejection of paternal antigens. The major risk in late pregnancy is vertical transmission, while in early pregnancy infection may cause congenital malformations, abortion, and intrauterine growth restrictions [6]. Despite adequate antiviral treatment, maternal mortality has been estimated at 20% to 30% [3].

## 2. Case Presentation

**Figure 2:**
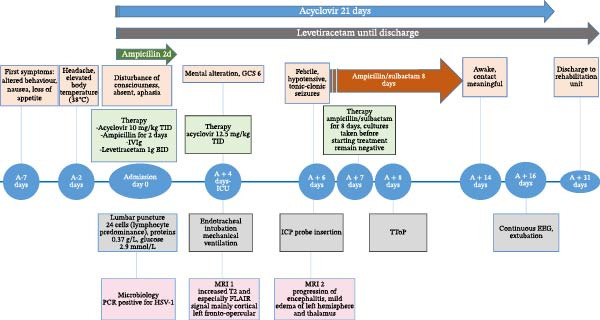
Timeline of the clinical course. A, admission; BID, twice daily; CRP, C‐reactive protein; CSF, cerebrospinal fluid; GCS, Glasgow Coma Scale; HSV, herpes simplex virus; ICP, intracranial pressure; ICU, intensive care unit; IVIgs, intravenous immunoglobulins; MRI, magnetic resonance imagining; PCR, polymerase chain reaction; TID, three times daily.

A 32‐year old right‐handed woman at 12 weeks of gestation presented to the Emergency Department of the University Medical Center Ljubljana, Slovenia, with febrile illness and disturbance of consciousness. This was her third pregnancy. She had previously undergone one termination of pregnancy and had delivered a healthy newborn at term after an uncomplicated pregnancy. The current pregnancy had been uneventful until the onset of acute symptoms in the 12^th^ week of gestation. Relatives reported behavioral changes had begun 7 days prior to the emergency department visit. She had complaint of headache, nausea, and loss of appetite. Upon admission, she appeared unresponsive, aphasic, and had a fixed gaze. A new‐onset right‐sided paresis was also observed. After brain magnetic resonance imaging (MRI), lumbar puncture was performed. Cerebrospinal fluid (CSF) analysis showed pleocytosis (24 × 10^6^/L, predominantly lymphocytes), proteins 0.37 g/L, and a normal CSF‐to‐blood glucose ratio (0.47). Empirical treatment with acyclovir 10 mg/kg three times daily (TID) and ampicillin. Ampicillin was discontinued after CFS polymerase chain reaction (PCR) testing confirmed HSV‐1. Intravenous immunoglobulin therapy was initiated but discontinued due to pyrexia and torticollis during the infusion. While hospitalized at the neurology ward she had two tonic‐clonic seizures and received levetiracetam 1 g twice daily (BID). Follow‐up MRI demonstrated unspecific changes (Figure 1A,B). On Day 4, the patient became comatose and required intubation and mechanical ventilation. She was transferred to the ICU of the Department of Infectious Diseases. The acyclovir dose was increased to 12.5 mg/kg TID. Due to the onset of recurrent clonic seizures on Day 6, a repeat MRI was performed, demonstrating progression of inflammatory changes and mild diffuse cerebral edema (Figure 1C,D). An intracranial pressure (ICP) monitoring probe was inserted; however, all measurements remained within the normal range. The clinical course was further complicated by ventilator‐associated pneumonia of undetermined etiology, and empirical treatment with ampicillin/sulbactam was administered for 8 days. A multidisciplinary team meeting was convened. Therapeutic termination of pregnancy (TToP) was suggested because the pregnancy‐related immunocompromised state was considered to pose a significant risk to the mother’s life. Pregnancy was terminated with mifepristone and misoprostol, followed by uterine curettage due to uterine bleeding on Day 8. A follow‐up brain computer tomography (CT) scan showed no new changes. The ICP probe was removed, analgosedation was discontinued, and the patient awakened under continuous electroencephalographic monitoring. She was extubated on Day 16 and discharged on Day 31 to the rehabilitation unit. At discharge, she had residual motor aphasia, forming only short sentences, but she was able to feed herself and walk with the assistance of a crutch. According to current recommendations, she received acyclovir for 21 days [7]. Figure 2 presents the timeline of clinical, laboratory, radiological, and microbiological findings.

Figure 1(A and B) Brain MRI on Day 4—increased T2 and especially FLAIR signal mainly in the cortical left fronto‐opercular, in this area there is also an increased DWI signal and hypointensity—diffusion restriction—it could be fresh ischemia, changes after an epileptic seizure, or as part of inflammation. Small areas of increased FLAIR signal in the cortical left frontal and in the left cingulate gyrus also indicated opercular right minimal and left parietal; a small area of increased signal on T2 and FLAIR sequence without clear changes on DWI sequence is also visible in the medial part of the right thalamus. (C and D) Brain MRI on Day 6—progression of encephalitic lesions compared to previous examination. Focal edema is present in areas of inflammation. Cerebrospinal spaces are slightly narrower than previously, possibly mainly due to areas of focal edema, with mild diffuse edema also possible, no pronounced diffuse edema is seen.

**Figure:**
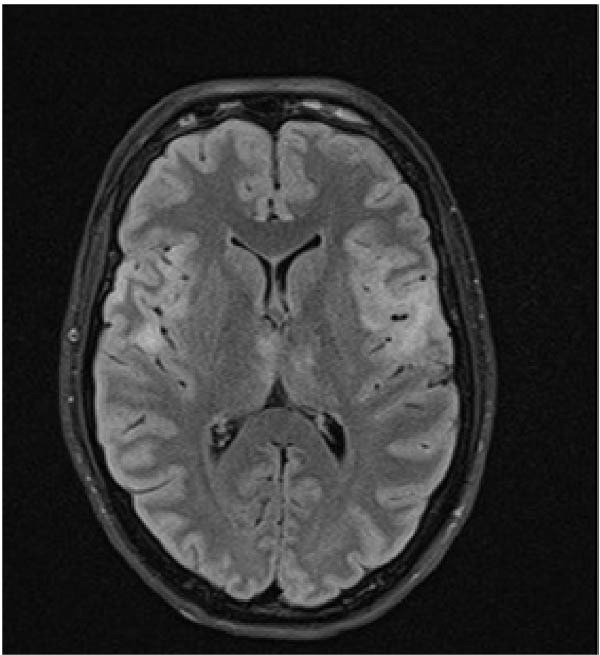
(A)

**Figure:**
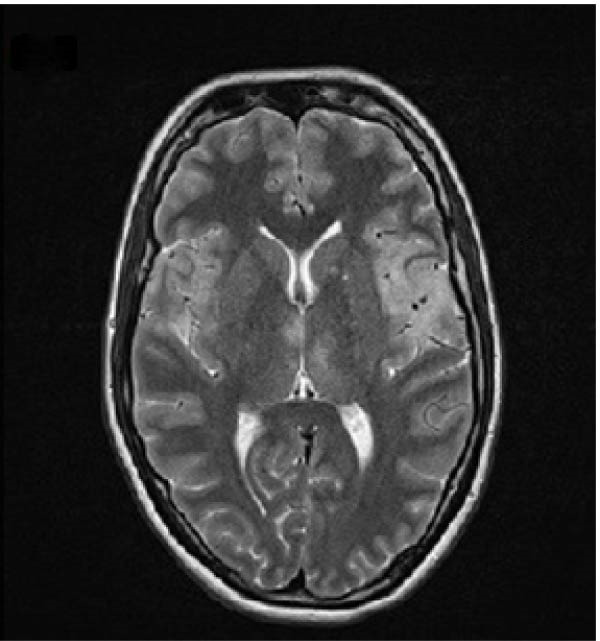
(B)

**Figure:**
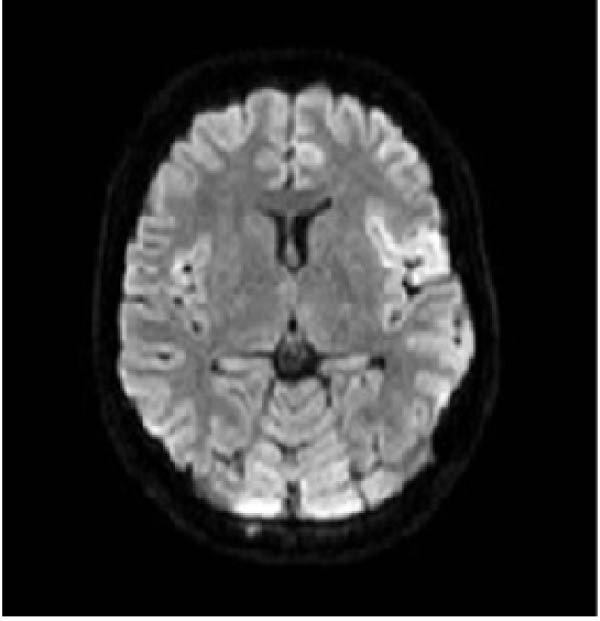
(C)

**Figure:**
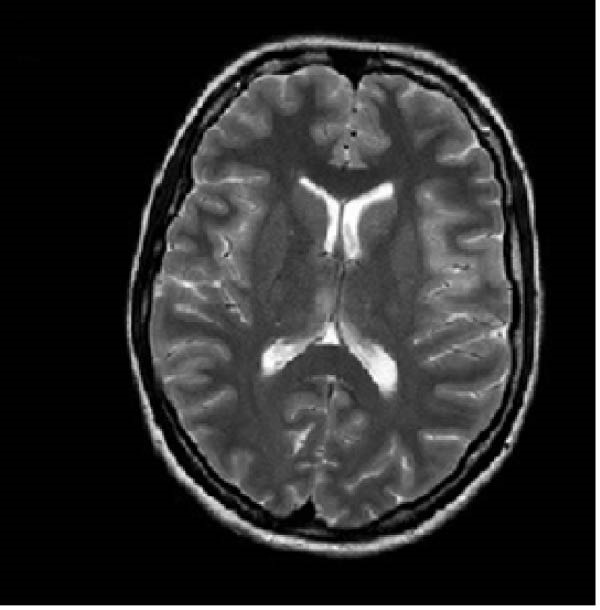
(D)

The patient’s written consent was obtained.

## 3. Discussion

Although the patient received appropriate antiviral treatment, disease progression ceased only after TToP, a procedure that can be used if the mother’s life/health is endangered [8]. We hypothesize that pregnancy‐related immunosuppression contributed to the progression of inflammation in this case. However, this remains speculative since we present a single case of HSVE in the first trimester of pregnancy. Furthermore, HSVE in young healthy immunocompetent nonpregnant women has not been described in peer‐reviewed literature yet. Therefore, no firm conclusions can be drawn regarding differences in HSVE course associated with pregnancy. Nevertheless, our case suggests that worsening HSVE in early pregnancy despite adequate antiviral and supportive treatment may warrant consideration of TToP to prevent further maternal morbidity and potentially mortality.

Our patient suffered severe neurological sequelae. Studies have shown that extensive MRI brain changes [9], early onset of epileptic seizures [10] and delayed initiation of antiviral therapy [2] are associated with poor functional outcome, which may explain unfavorable course in the case presented.

Most guidelines recommend an acyclovir dose of 10 mg/kg TID; however, our patient received higher dose [7]. Pouissier et al. [2] recently reported no influence of high dose acyclovir (15 mg/kg TID) on neurological outcome in nonpregnant individuals. Volume of distribution for hydrophilic and hydrophobic drugs is increased in pregnancy, which results in lower plasma concentrations. Acyclovir has poor solubility in lipophilic and hydrophilic environments and due to physiological adaptations in pregnancy, its concentration decreases even more [11]. Similar pharmacokinetic changes occur during childhood; consequently, pediatric doses up to 20 mg/kg TID might be used [12, 13].

Invasive ICP measurement is not routinely recommended in encephalitis management but was necessary in our case due to epileptic seizures and the presence of cerebral edema [14].

HSVE rarely occurs in early pregnancy, but when it does, its course may be severe due to pregnancy‐related immune changes. In this case, treatments not routinely recommended in HSVE management were used, including ICP monitoring and high‐dose antivirals. As the patient’s condition continued to deteriorate despite appropriate treatment, TToP was performed. Improvement may have resulted from a combination of factors rather than a single intervention, including delayed therapeutic effect of antiviral treatment, the impact of intensive care management and supportive neurocritical care, seizure control, optimization of antiepileptic therapy, or the possibility of spontaneous recovery as part of the natural disease course. Nevertheless, this case highlights the importance of multidisciplinary collaboration and individualized judgment in rare and complex situations. TToP presents an ethical dilemma requiring clinicians to balance maternal autonomy, fetal considerations, and the duty to minimize harm in the context of uncertain prognosis, emphasizing individualized decision‐making and shared counseling.

## Author Contributions

**Nina Grasselli Kmet:** conceptualization, methodology, visualization, investigation, writing – reviewing and editing, supervision. **Matej Furlan**: investigation, visualization, data curation, writing – original draft preparation, software. **Miha Lučovnik:** writing – reviewing and editing, supervision.

## Funding

The authors have nothing to report.

## Ethics Statement

A patient’s written consent was obtained to publish this report.

## Conflicts of Interest

The authors declare no conflicts of interest.

## Data Availability

The data that support the findings of this study are available from the corresponding author upon reasonable request.
